# Differential response of hippocampal and prefrontal oscillations to systemic LPS application

**DOI:** 10.1016/j.brainres.2017.12.036

**Published:** 2018-02-15

**Authors:** Omar Mamad, Md Nurul Islam, Colm Cunningham, Marian Tsanov

**Affiliations:** a Trinity College Institute of Neuroscience, Trinity College Dublin, Ireland; b School of Psychology, Trinity College Dublin, Ireland; c School of Biochemistry and Immunology, Trinity College Dublin, Ireland; d Trinity Biomedical Sciences Institute, Trinity College Dublin, Ireland

**Keywords:** LPS, Lipopolysaccharide, mPFC, Medial prefrontal cortex, LFP, Local field potential, SWD, Spike-wave discharges, EEG, Electroencephalogram, LPS, Hippocampus, Medial prefrontal cortex, Theta, Delta

## Abstract

The early electrophysiological phenomena linked to systemic inflammation are largely underexplored. We developed here local field analyses to detect prodromal oscillatory abnormalities. We identified early band-specific patterns in local field potential recorded from freely-moving rats injected intraperitoneally with lipopolysaccharide (LPS, 1 mg/kg). Theta frequency was significantly reduced and this effect was not related to the decreased locomotion of the animal. Furthermore, LPS-induced alterations show a region-specific response when compared between the hippocampal region and medial prefrontal cortex. Delta mean frequency increased in the hippocampal region but not in the prefrontal cortex. We explored also the hypothesis that systemic inflammation increases the propensity of abnormally synchronized brain activity. Our data indicate that the LPS-evoked alteration of delta and theta frequency parameters reflects the formation of abnormal synchronization in similar frequency ranges. The onset of abnormal brain activity was indicated by spike-wave discharges in the range of 1–10 Hz with three main frequency domains. Importantly, the occurrence of spike-wave discharges was observed in the hippocampus but not in the cortex. In summary, the hippocampal theta rhythm is an accurate indicator of the oscillatory changes evoked by LPS application. The findings offer clear patterns of altered brain function that will facilitate mechanistic investigations of brain dysfunction and delirium occurring during sepsis.

## Highlights

• 1 mg/kg LPS i.p. injection robustly suppresses theta frequency in hippocampus.
• LPS administration augments delta frequency in hippocampus but not mPFC.
• LPS injection triggers hippocampal spike-wave discharges.

## 1 Introduction

Sepsis is one of the leading causes of mortality in intensive care units. The identification of brain dysfunction in the early stages in sepsis is a significant challenge because there are no instant biomarkers of neuronal injury. Furthermore, the evaluation of cognitive performance does not reflect the degree of neuronal attenuation in the onset of systemic inflammation (Zampieri et al., 2011). Electroencephalogram (EEG) abnormalities occurring at the acute stage of sepsis may correlate with severity of brain dysfunction. Predictive value of early standard EEG abnormalities for mortality in septic patients remains to be assessed. We know that generalized slowing on EEG is one of the main signs of septic encephalopathy (Young et al., 1990). Delirium is a major component of the clinical presentation of sepsis-associated encephalopathy and its states have been characterized with deceleration or loss of cortical rhythms, global slowing and intermittent rhythmic delta activity (Jacobson and Jerrier, 2000, Klass and Brenner, 1995). Delirium evokes dissimilar effects on the frequency and power of the brain rhythms. A decrease in alpha but an increase in delta power was observed in delirium patients (Koponen et al., 1989). Delirium is often measured in patients with coexisting neurodegenerative pathophysiology. The EEG in dementia also reveals generalized slowing of cortical oscillations, although this slowing is less consistent as compared to that in delirium (Engel and Romano, 2004). The investigation of other frequency ranges might increase the specificity of oscillation profile in sepsis-affected patients. An activated high alpha and delta spectral power density, increased the diagnostic correctness and specificity of detecting delirium patterns in patients with dementia (Thomas et al., 2008). To assess the ability of animal models to replicate key aspects of delirium and to obtain more specific electrophysiological markers of inflammation-triggered delirium we need intracortical recordings from animal models of early systemic inflammation that will serve as a direct measure of prodromal brain dysfunction. There is a limited information on the effects of systemic inflammation on local field potential (LFP) in behaving animals. In animals with Biperiden-induced hypoactive delirium state delta and low theta are significantly increased, while the high theta power values are significantly decreased compared to controls (Tamura et al., 2006). A decrease in overall spectral frequency with suppression of alpha and augmentation of delta power was shown in LPS-induced delirium animal model (Semmler et al., 2008). Rats injected with high dose of LPS also display epileptiform pathophysiology of brain oscillations. LPS-induced inflammatory response increases the number of spike-wave discharges in rats in a dose dependent manner (Kovacs et al., 2006). The medial temporal lobe is the most typical area of seizure initiation and the hippocampal region is a central to the generation of epileptiform discharges (Avoli et al., 2002). Therefore, the LFP evaluation in acute LPS-induced encephalopathy should be optimised by the measurement of region-specific, frequency-specific and discharge-specific spectral analysis. Running speed influences both the power and frequency of theta oscillations seen in the hippocampal LFP (Geisler et al., 2007, McFarland et al., 1975, Shin and Talnov, 2001). Similarly, gamma oscillations shift to higher frequencies at faster speeds (Ahmed and Mehta, 2012). Another study in behaving mice found that both low and high gamma power were found to increase as a function of running speed in mice (Chen et al., 2011). However, up to now the studies investigating the effect of systemic inflammation on EEG did not account for the whole-body speed of the animals in of their inflammation-evoked LFP evaluations. We investigated here the intracortical LFP changes in hippocampal and prefrontal cortices of LPS-injected rats as a function of whole-body speed. We used a moderate dose of LPS to determine whether the early stages of brain dysfunction after systemic inflammation can be assessed by LFP parameters. To increase diagnostic accuracy we performed time-dependent spectral analysis of the hippocampal and prefrontal LFP that was evaluated 1.5, 3, 4, 6, 8 and 24 h after the LPS administration.

## 2 Results

### 2.1 LPS robustly decreases hippocampal theta frequency

**Fig. 1:**
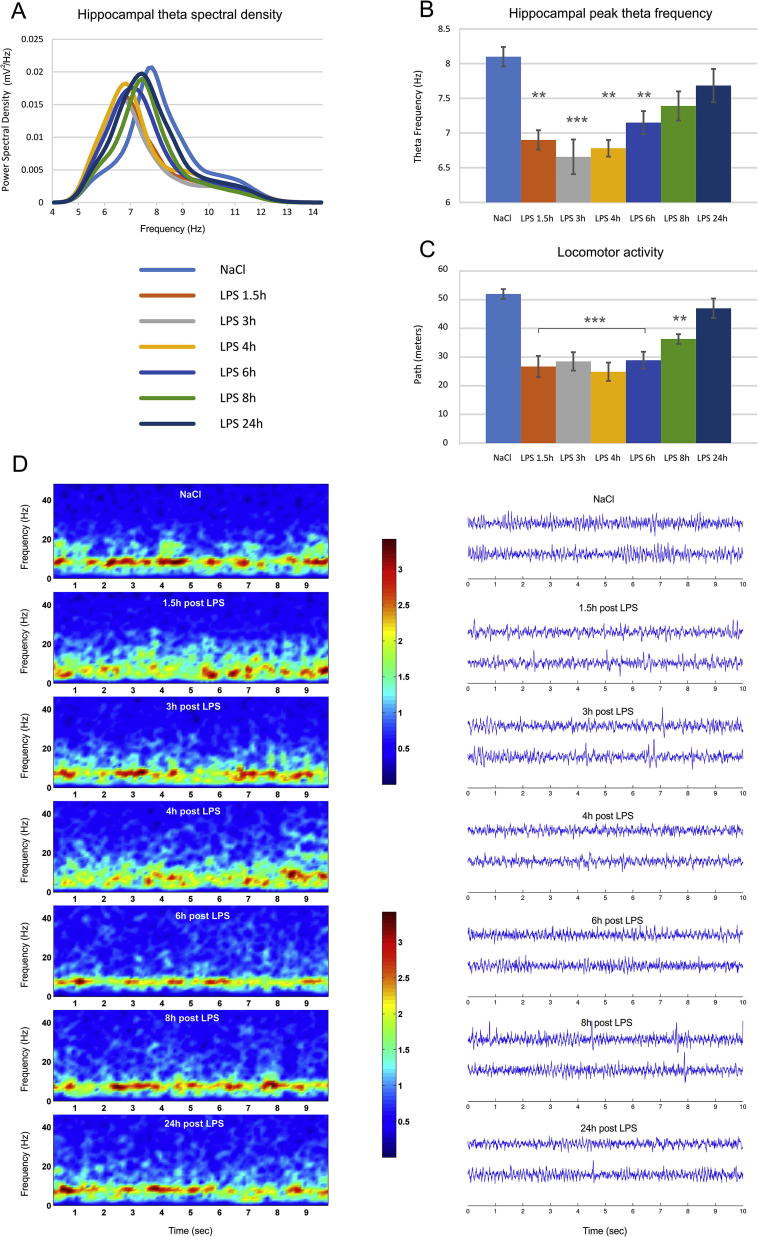
LPS administration decreases theta oscillations in hippocampus and whole-body speed of behaving rats. (A) Theta power spectral density visualized for control saline injection (light blue) 24 h prior the LPS injection, 1.5 h after injection of lipopolysaccharide (LPS, 1 mg/kg) (orange), 3 h (grey), 4 h (yellow), 6 h (blue), 8 h (green) and 24 h (dark blue). (B) Peak hippocampal theta frequency (Hz) for controls and LPS injected animals. Bonferroni post hoc test ^***^P < .001, ^**^P < .01. (C) Covered path (meters) per recording session for controls and LPS injected animals. Bonferroni post hoc test ^***^P < .001, ^**^P < .01. Error bars, mean ± s.e.m. (D) 10 s epochs of color-coded power spectrogram (left panels) and two LFP traces (right panels) recorded from hippocampus in control (top) and LPS-injected animals (below) 1.5, 3, 4, 6, 8 and 24 h after the injection of LPS.

Baseline recordings with saline injection estimated the spectral power of the local filed potential (LFP) recorded from Lister Hooded rats. 24 h later we injected the same rats with LPS i.p. (1 mg/kg) and measured the local filed potential (LFP) in the CA1 region of hippocampus (n = 6) and medial prefrontal cortex (n = 6). We evaluated the changes of the power spectral density 1.5 h, 3 h, 4 h, 6 h, 8 h and 24 h after the LPS injection. The power spectral density of theta range (5–12 Hz) in hippocampus (Fig. 1A and D) showed frequency decrease after the LPS injection, which recovered gradually in the next 24 h. The peak theta frequency (Fig. 1B) decreased significantly for 6 h after the LPS injection from 8.07 Hz in the baseline recording to 6.89 Hz 1,5 h, 6.65 Hz 3 h, 6.78 Hz 4 h and 7.15 Hz 6 h post-LPS (one-way ANOVA with Bonferroni post hoc test, between groups, n = 6, F_(6,41)_ = 7.204, *P* < .001). The frequency of theta rhythm is positively correlated with the speed (Shin and Talnov, 2001) thus, the decrease of theta frequency could be related to the reduced locomotor activity of the animals after the LPS injection. LPS induced significant suppression in locomotor activity from 52.02 ± 8.2 m per 12 min recording session to 26.71 ± 10.2 m at 1.5 h after the injection (one-way ANOVA with Bonferroni post hoc test, between groups, n = 6, F_(6,41)_ = 9.978, *P* < .001) and 24 h later recovered to 47.1 ± 9.9 m (Fig. 1C). Therefore, the observed reduction in the oscillatory rate (Fig. 1D) cannot be evaluated independently from the speed of the animals.

**Fig. 2:**
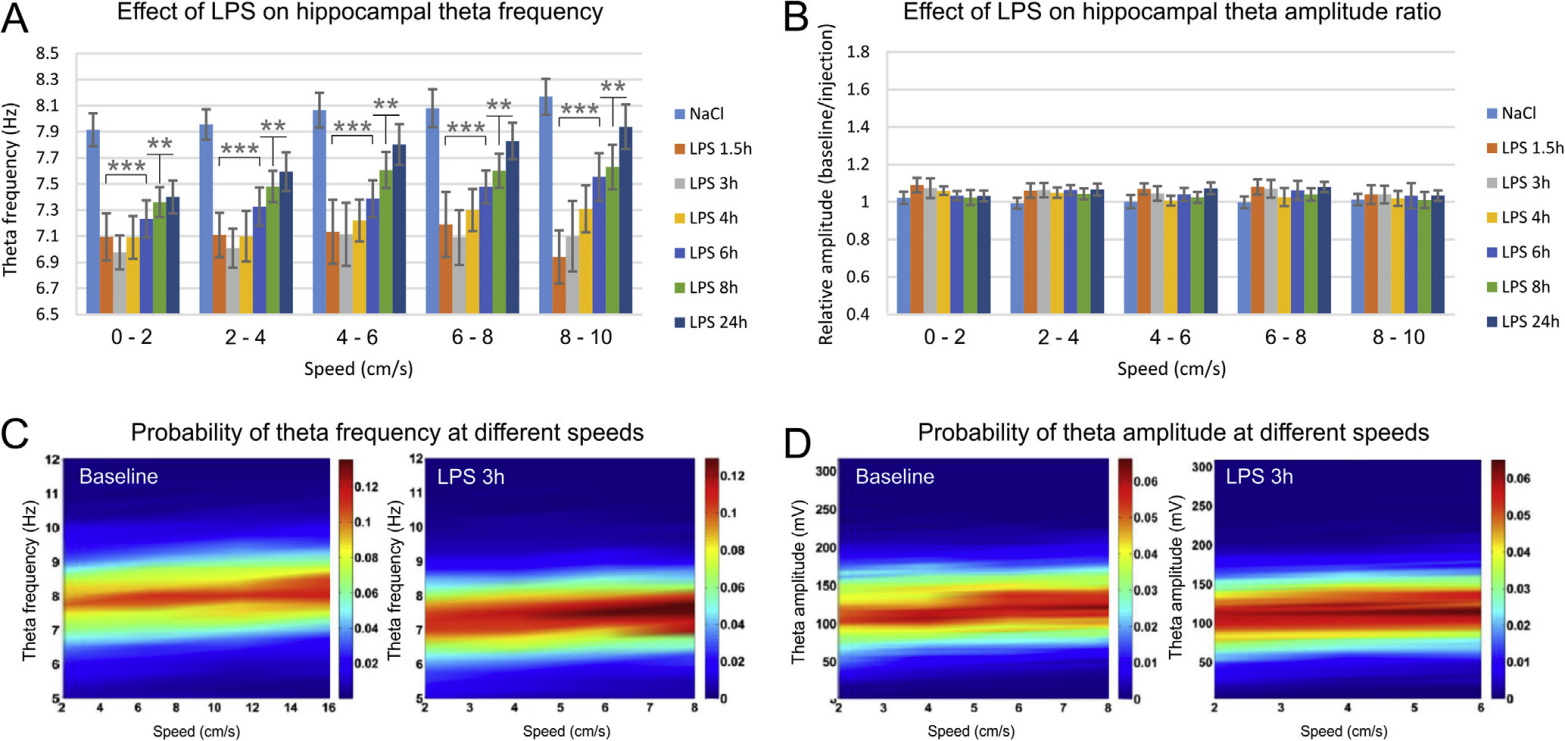
LPS-induced decrease of hippocampal frequency but not amplitude. (A) Hippocampal theta frequency visualized for control saline injection (light blue), 1.5 h after LPS injection (orange), 3 h (grey), 4 h (yellow), 6 h (blue), 8 h (green) and 24 h (dark blue). The results are grouped in speed-bands of 2 cm/s: 0–2 cm/s, 2–4 cm/s, 4–6 cm/s, 6–8 cm/s, 8–10 cm/s. Bonferroni post hoc test ^***^P < .001, ^**^P < .01. Error bars, mean ± s.e.m. (B) Relative hippocampal theta amplitude measured with the ratio of baseline over injection recording for different speed ranges. (C) Color-coded spectrograms of the probability of theta frequency at different speeds. Left panel shows representative probability spectrogram from the baseline recording, while the right probability spectrogram – from recording 3 h after the LPS administration. (D) Color-coded spectrograms of the probability of theta amplitude at different speeds. Left probability spectrogram: baseline recording; right – 3 h post LPS injection recording.

To test the hypothesis that the reduced theta frequency is not simply a result from the decreased locomotion of the animal we have analysed theta oscillations for five low speed ranges: 0–2 cm/s, 2–4 cm/s, 4–6 cm/s, 6–8 cm/s and 8–10 cm/s. The post-injection decrease of theta frequency was significant for all speed sub-ranges (two-way ANOVA with Bonferroni post hoc test, between groups, n = 6, F_(6,209)_ = 19.620, *P* < .001) with consistent effect in the first 8 h after the LPS injection (Fig. 2A and C). Concurrently, we observed no significant change in the amplitude of theta rhythm and this was evident in the comparison between the baseline and LPS-recordings for the low speed ranges (Fig. 2B and D, two-way ANOVA with Bonferroni post hoc test, between groups, n = 6, F_(6,209)_ = 1.235, *P* = .290).

### 2.2 LPS diminishes mean frequency and amplitude of prefrontal theta rhythm

**Fig. 3:**
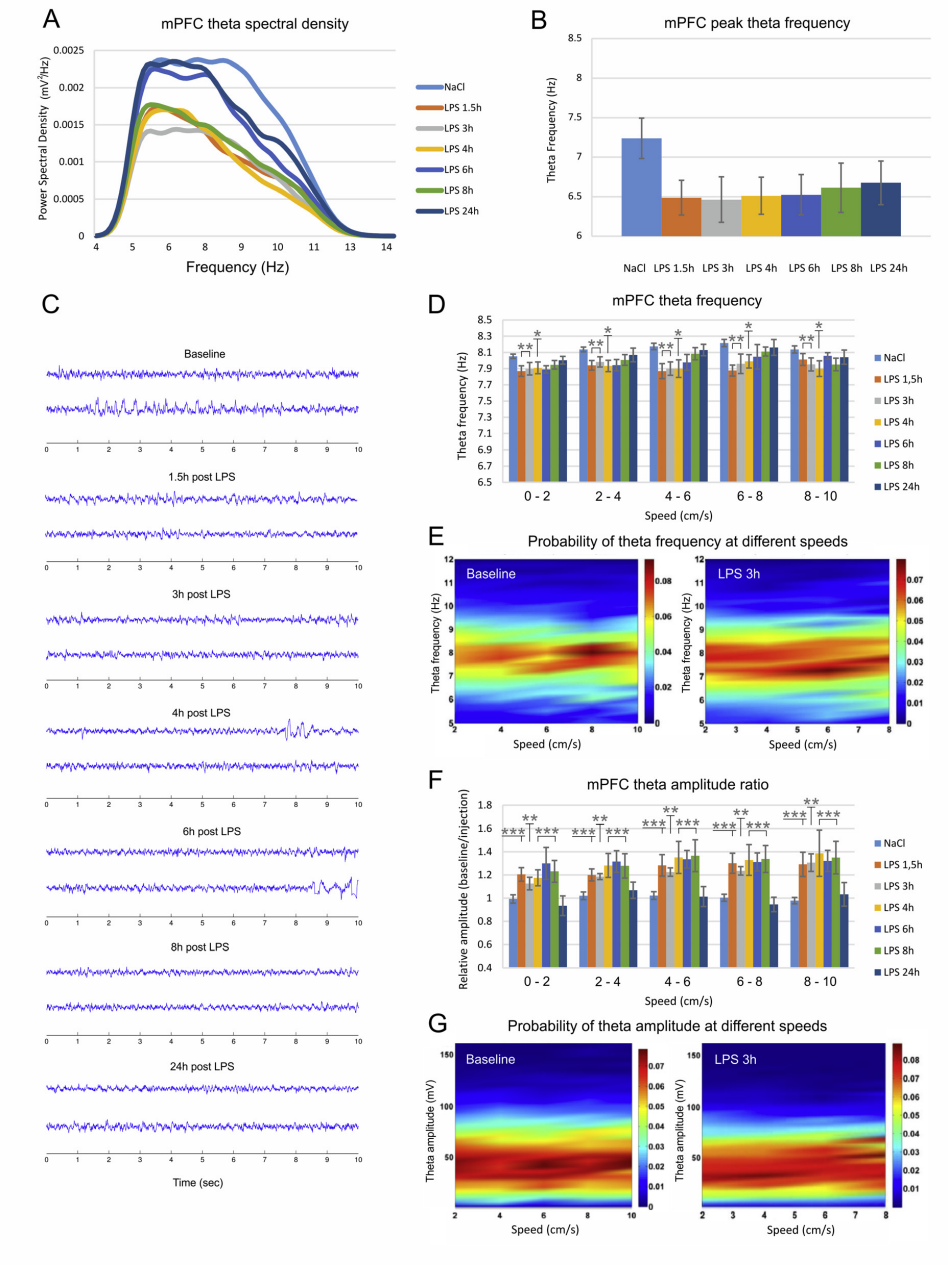
LPS-induced decrease of prefrontal frequency and amplitude. (A) Theta power spectral density recorded from the medial prefrontal cortex (mPFC) visualized for control saline injection (light blue), 1.5 h after LPS injection (orange), 3 h (grey), 4 h (yellow), 6 h (blue), 8 h (green) and 24 h (dark blue). (B) Peak prefrontal theta frequency (Hz) for controls and LPS injected animals. (C) 10 s epochs of two LFP traces recorded from mPFC in control (top) and LPS-injected animals (below) 1.5, 3, 4, 6, 8 and 24 h after the injection of LPS. (D) mPFC theta frequency visualized for control saline injection (light blue), 1.5 h after LPS injection (orange), 3 h (grey), 4 h (yellow), 6 h (blue), 8 h (green) and 24 h (dark blue). The results are grouped in speed-bands of 2 cm/s: 0–2 cm/s, 2–4 cm/s, 4–6 cm/s, 6–8 cm/s, 8–10 cm/s. Bonferroni post hoc test ^**^P < .01, ^*^P < .05, (E) Color-coded spectrograms of the probability of theta frequency at different speeds. Left panel shows representative probability spectrogram from the baseline recording, while the right probability spectrogram – from recording 3 h after the LPS administration. (F) Relative mPFC theta amplitude measured with the ratio of baseline over injection recording for different speed ranges. Bonferroni post hoc test ^***^P < .001, ^**^P < .01. Error bars, mean ± s.e.m. (G) Color-coded spectrograms of the probability of theta amplitude at different speeds. Left probability spectrogram: baseline recording; right – 3 h post LPS injection recording.

In another group of animals we evaluated the effect of LPS injection on theta rhythm in medial prefrontal cortex (mPFC). The control power spectral density of theta range (5–12 Hz) expressed different profile from the hippocampus where there was a clear peak around 8 Hz. In the prefrontal cortex theta rhythm shows similar power density in the range of 5–9 Hz (Fig. 3A). LPS application reduced the spectral power of the upper theta and reduced the peak of theta frequency (Fig. 3B) from 7.23 Hz in the baseline recording to 6.48 Hz at 1,5 h after the LPS injection. This effect was not significant (one-way ANOVA with Bonferroni post hoc test, between groups, n = 6, F_(6,41)_ = 1.032, P = .421), showing that LPS injection had much smaller effect on prefrontal theta peak frequency (Fig. 3C) compared to hippocampus. However, the LPS effect on prefrontal mean theta frequency for the low speed ranges (Fig. 3D and E) showed significant differences in the first 4 h after the injection (two-way ANOVA with Bonferroni post hoc test, between groups, n = 6, F_(6,209)_ = 4.228, P < .001). We next evaluated the amplitude change of mPFC theta oscillations. We evaluated the relative amplitude ratio measured as the ratio of the absolute amplitude from the baseline recording over the absolute amplitude from the injection recording. The LPS injection significantly reduced theta amplitude up to 8 h after the injection (expressed by an increase of the relative theta amplitude ratio, Fig. 3F and G), adding another difference to the hippocampal response. The relative theta amplitude ratio significantly increased 1.5 h after the injection to 1.20 ± 0.14 for 0–2 cm/s; 1.20 ± 0.12 for 2–4 cm/s; 1.28 ± 0.22 for 4–6 cm/s; 1.30 ± 0.21 for 6–8 cm/s; 1.29 ± 0.25 for 8–10 cm/s (two-way ANOVA with Bonferroni post hoc test, between groups, n = 6, F_(6,209)_ = 12.314, *P* < .001). The relative theta amplitude ratio returned to was back to baseline levels 24 h later (Fig. 3F).

**Fig. 4:**
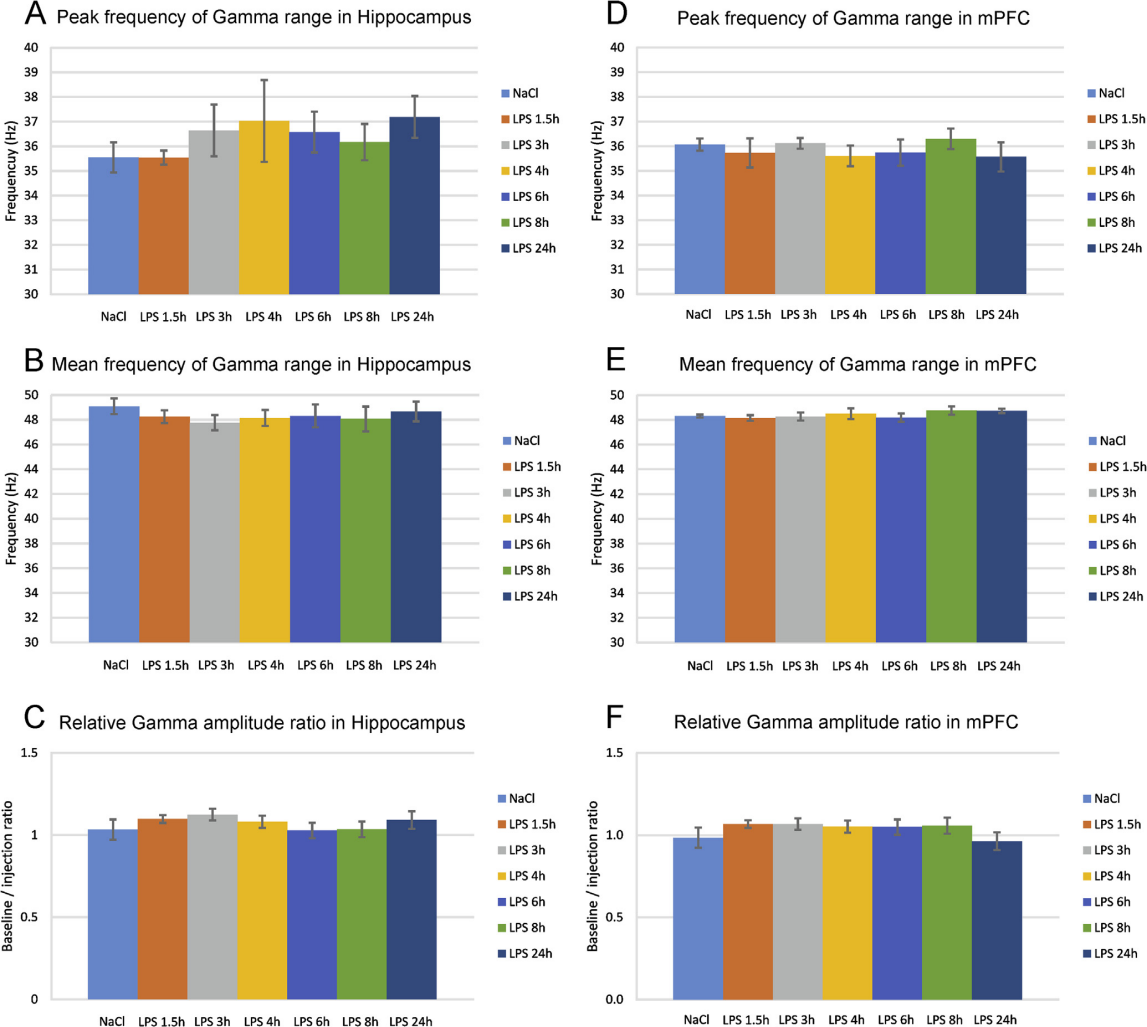
Systemic inflammation does not evoke significant effect on gamma oscillations. (A) Peak hippocampal gamma frequency (Hz) for controls and LPS injected animals. (B) Mean hippocampal gamma frequency for controls and LPS injected animals. (C) Relative hippocampal gamma amplitude measured with the ratio of baseline over injection recording. Peak (D) and mean (E) prefrontal gamma frequency for controls and LPS injected animals. (F) Relative mPFC gamma amplitude.

The alterations of theta rhythm are often paralleled by concurrent changes of high frequency oscillations (gamma range, 40–100 Hz) particularly in the hippocampal formation (Colgin, 2015). Here, we found no significant change of gamma peak (Fig. 4A) and mean (Fig. 4B) frequency as well as gamma amplitude (Fig. 4C) in hippocampus. Similarly, the LPS injection had no effect on mPFC gamma peak frequency (Fig. 4D), mean frequency (Fig. 4E) and amplitude (Fig. 4F). These findings show that low frequencies are more accurate indicator of the oscillatory changes evoked by LPS application compared to high frequencies. Furthermore, hippocampal and prefrontal LPS-induced alterations have differential profile, showing a region specific and frequency-specific response to systemic inflammation induced by *E. coli* LPS.

### 2.3 LPS evokes significant changes only to the mean delta frequency in hippocampus

**Fig. 5:**
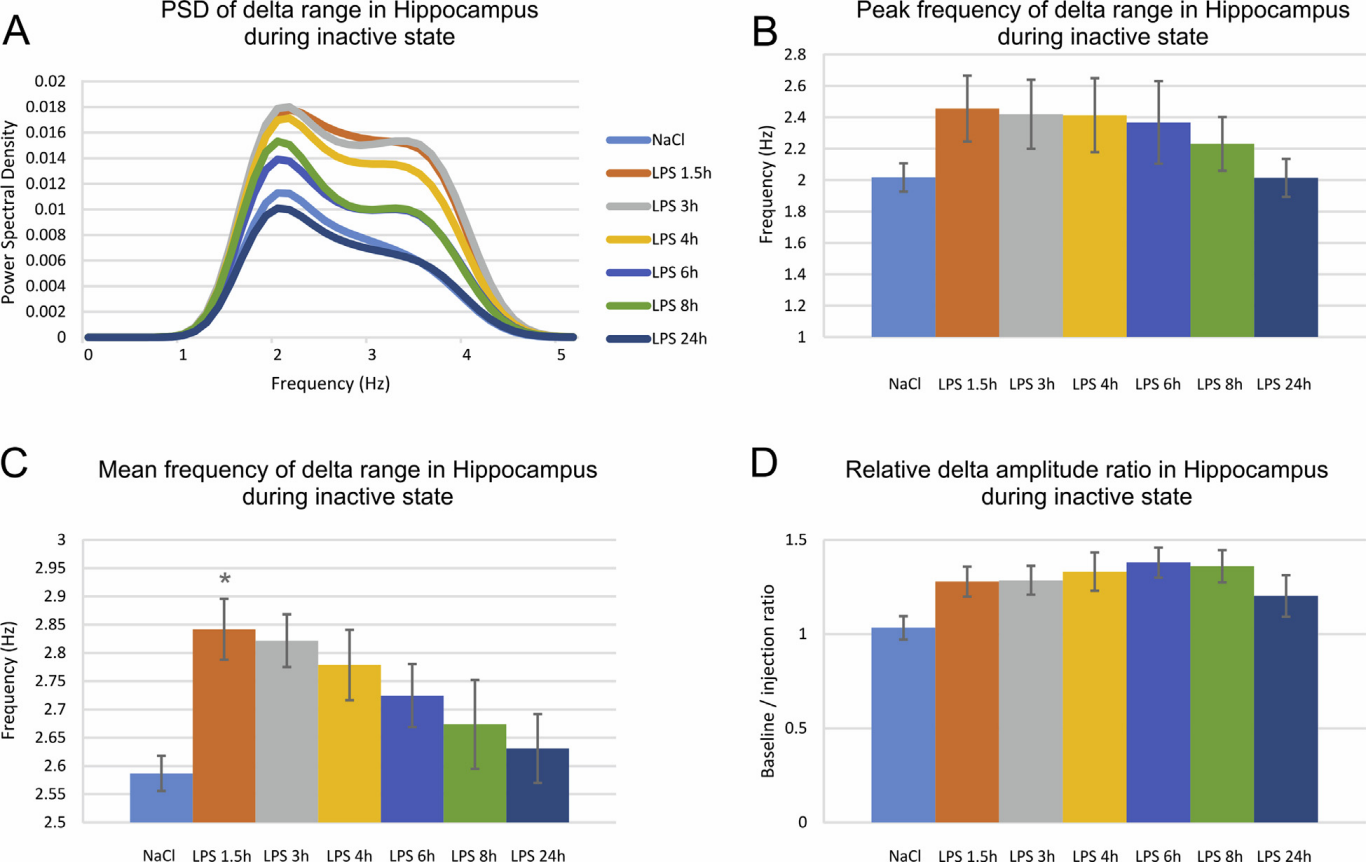
LPS-induced short-term decrease of hippocampal mean delta frequency. (A) Hippocampal delta power spectral density visualized for control saline injection (light blue), 1.5 h after LPS injection (orange), 3 h (grey), 4 h (yellow), 6 h (blue), 8 h (green) and 24 h (dark blue). (B) Peak and (C) mean hippocampal delta frequency (Hz) for controls and LPS injected animals. (D) Relative hippocampal delta amplitude measured with the ratio of baseline over injection recording. Bonferroni post hoc test ^*^P < .05. Error bars, mean ± s.e.m.

We examined the effect of LPS injection on another low frequency range, i.e. delta (1–4 Hz). Slower oscillations are distinctive feature of the thalamic-cortical networks during quiet waking (Steriade et al., 1993). Thus, we evaluated delta power and frequency during immobility (quite waking) for the control and LPS-injected animals. Again, we compared the delta parameters for hippocampus and mPFC. Hippocampal low filtered power spectral density showed increase of the upper delta range (Fig. 5A). The peak frequency showed only a non-significant tendency for increase (Fig. 5B) with shift from 2.01 ± 0.23 Hz to 2.45 ± 0.51 at 1.5 h post-LPS, 2.41 ± 0.53 at 3 h post-LPS and 2.41 ± 0.57 at 4 h post-LPS (one-way ANOVA with Bonferroni post hoc test, between groups, n = 6, F_(6,41)_ = 0.946, *P* = .474). Concurrently, the mean delta frequency significantly increased 1.5 h after the LPS injection from 2.58 ± 0.08 to 2.84 ± 0.13 (one-way ANOVA with Bonferroni post hoc test, between groups, n = 6, F_(6,41)_ = 2.828, *P* = .024). The tendency for increase continued for the next two subsequent recordings: 2.82 ± 0.11 Hz 3 h post-LPS and 2.77 ± 0.15 Hz 4 h post-LPS (Fig. 5C). The amplitude of hippocampal delta slightly decreased up to 8 h after the injection, expressed by increase of the relative amplitude ratio (1.28 ± 0.26 at 1.5 h post-LPS; for 1.28 ± 0.18 at 3 h post-LPS; 1.33 ± 0.24 at 4 h post-LPS; 1.38 ± 0.25 at 6 h post-LPS and 1.36 ± 0.21 at 8 h post-LPS). The changes of the amplitude values, however, were not statistically significant (Fig. 5D, one-way ANOVA with Bonferroni post hoc test, between groups, n = 6, F_(6,41)_ = 1.515, *P* = .202).

**Fig. 6:**
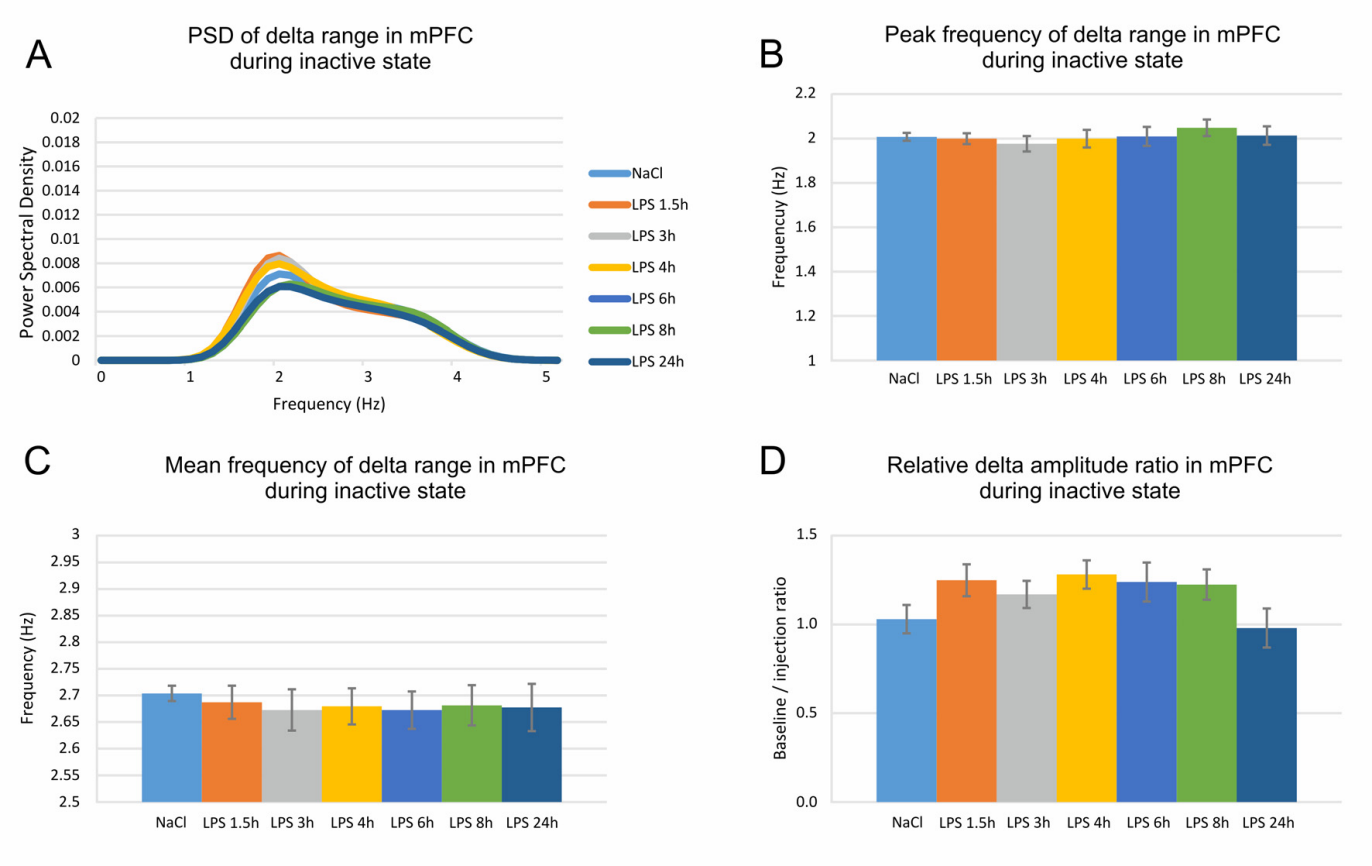
LPS has no effect on prefrontal delta rhythm. (A) Prefrontal delta power spectral density visualized for control saline injection (light blue), 1.5 h after LPS injection (orange), 3 h (grey), 4 h (yellow), 6 h (blue), 8 h (green) and 24 h (dark blue). (B) Peak and (C) mean prefrontal delta frequency (Hz) for controls and LPS injected animals. (D) Relative prefrontal delta amplitude measured with the ratio of baseline over injection recording.

We found no shift of the peak delta frequency in the mPFC recordings (Fig. 6A and B; one-way ANOVA with Bonferroni post hoc test, between groups, n = 6, F_(6,41)_ = 0.455, *P* = .837). While the mean hippocampal delta frequency increased, we found no slow frequency change in the mPFC recordings. The mean delta mPFC frequency ranged from 2.70 ± 0.08 in the baseline recording to 2.68 ± 0.07 at 1.5 h post-LPS (Fig. 6C, one-way ANOVA with Bonferroni post hoc test, between groups, n = 6, F_(6,41)_ = 0.142, *P* = .990). The amplitude of mPFC delta decreased slightly (with increased relative amplitude ratio) but this change was not significant (Fig. 6D, one-way ANOVA with Bonferroni post hoc test, between groups, n = 6, F_(6,41)_ = 1.054, *P* = .408).

### 2.4 LPS triggers epileptiform discharges in hippocampus

**Fig. 7:**
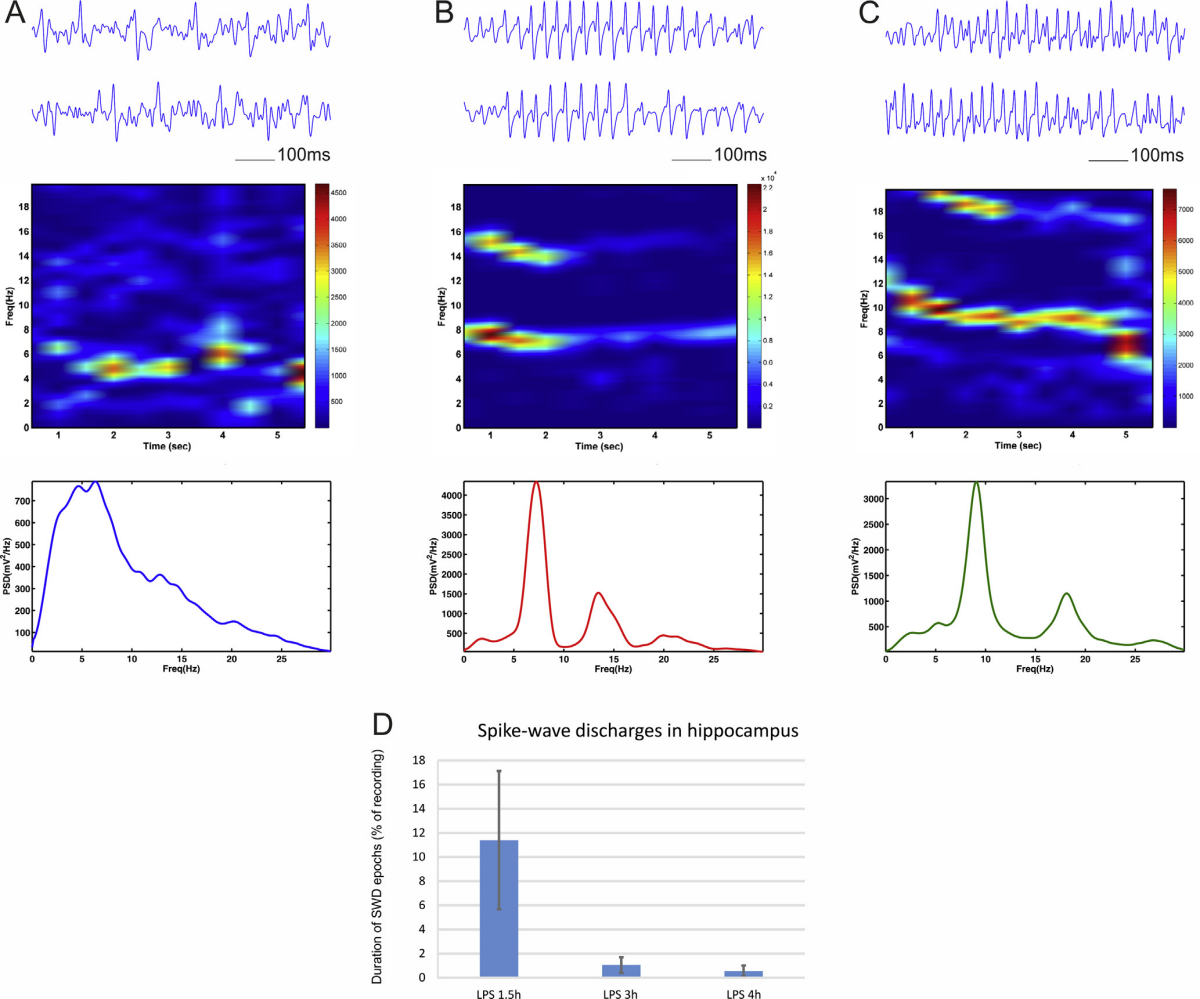
Spike-wave discharges generation in hippocampus after LPS administration. Top panels show LFP traces with slow frequency (A), intermediate- (B) and fast frequency (C) spike-wave discharges recorded from hippocampus in animals 1.5 h after intraperitoneal injection of lipopolysaccharide (LPS, 1 mg/kg). Middle panels show color-coded frequency spectrogram of the above epochs, while the panels below show power spectrum of the spike-wave discharges. (D) Duration of the spike wave discharges (SWD) as percent of baseline recording evaluated in recordings from 1.5, 3 and 4 h after the injection of LPS.

We also investigated the effect of low dose (1 mg/kg) LPS injection on the spike-wave discharges (SWD) in the local field oscillations. For the hippocampal recordings we observed the occurrence of pathological synchronization in 4 out of 5 animals. We identified the abnormal oscillatory patterns and analysed these oscillatory episodes separately from the rest of the recorded signal. The frequency profile of the spike-wave discharges showed three main types. 1) A slow SWD pattern in the range of 1–6 Hz, characterized with no clear peak (Fig. 7A); 2) an intermediate SWD pattern with peak of 5–7 Hz (Fig. 7B); and 3) a fast SWD peak rate in the range of 7–10 Hz (Fig. 7C). Both intermediate and fast SWDs showed harmonic oscillations. The SWD were predominantly expressed 1.5 h after the injection where the duration of the synchronization patterns was on average 11.39% of the local filed recordings (Fig. 7D). Thereafter, the occurrence of the epileptiform oscillations was strongly reduced to 1.05% at 3 h post-LPS and 0.55% at 4 h post LPS. We detected no SWD in the subsequent recordings. Unlike the hippocampal response to LPS injection the medial prefrontal recordings showed no epileptiform activity. We found no evidence of SWD in the mPFC after the LPS injection for 24 h. These data indicate that LPS evokes abnormal synchronization with frequencies within the delta and theta ranges. Our findings demonstrate the differential effects induced by systemic administration of bacterial lipopolysaccharides in hippocampal and prefrontal regions (Table 1).

**Table 1:** Changes in delta (δ), theta (θ) and spike-wave discharges (SWD) in the hippocampus (HP) and medial prefrontal cortex (mPFC) after systemic administration of lipopolysaccharide.

Post-LPS recording	δ amp HP	δ freq HP	θ amp HP	θ freq HP	SWD HP	δ amp mPFC	δ freq mPFC	θ amp mPFC	θ freq mPFC	SWD PFC
1.5 h	↓ns	↑*	–	↓***	∼11%	↓ns	–	↓***	↓**	–
3 h	↓ns	↑ns	–	↓***	∼1%	↓ns	–	↓**	↓**	–
4 h	↓ns	↑ns	–	↓***	∼0.5%	↓ns	–	↓***	↓*	–
6 h	↓ns	–	–	↓***	–	–	–	↓***	↓ns	–
8 h	–	–	–	↓**	–	–	–	↓***	–	–
24 h	–	–	–	–	–	–	–	–	–	–

## 3 Discussion

There are few data on electrophysiological alternations during systemic inflammation and its early effect on brain activity. Here, we examined the field oscillatory correlates that underlie brain dysfunction during acute systemic inflammation. We used peripheral injection of the bacterial endotoxin component lipopolysaccharide (LPS) to mimic moderate acute sepsis. We found that the hippocampal theta frequency, but not amplitude, was significantly reduced and this effect was not related to the decreased locomotion of the animal. Concurrently, both theta frequency and amplitude diminished in the medial prefrontal cortex (mPFC). No significant changes were observed in the gamma frequency range for hippocampal and prefrontal recordings. Delta frequency showed short-term hippocampus-specific increase, while the amplitude was not affected for both hippocampus and mPFC. Finally, we detected spike-wave discharges in the hippocampus but not in the prefrontal cortex.

### 3.1 EEG in delirium/septic encephalopathy

Severe systemic inflammation can evoke delirium in healthy individuals, while milder inflammation is sufficient to trigger delirium in patients with existing brain vulnerability. Preceding neurodegenerative pathology, such as synaptic loss and microglial priming (Cunningham et al., 2009, Davis et al., 2015), enhances the susceptibility to the effect of LPS-triggered systemic inflammation and augments the working memory deficits of mice (Murray et al., 2012). The LPS application protocol has been widely used to evoke systemic inflammation with resulting neuroinflammation in rodents and this has been adapted to developing animal models of delirium (Culley et al., 2014, Davis et al., 2015). A deterioration of brain metabolic function during delirium is clinically expressed with a characteristic cognitive disturbance in humans (Folstein et al., 1975). For reliable identification of early systemic inflammation-induced encephalopathy, correct differential diagnosis and prodromal features detection of brain oscillations essential we need region-specific intracortical local field measurements. Here, using electrodes implanted in hippocampal formation and mPFC we found that theta amplitude was reduced in both hippocampus and mPFC. This is consistent with the established finding that delirium episodes attenuate low-frequency oscillations in the frontal cortex (Jacobson and Jerrier, 2000, Klass and Brenner, 1995). Theta frequency in our experiments was significantly reduced and this effect was not related to the decreased locomotion of the animal. We further, detected region-specific increase of mean delta frequency in hippocampus but not mPFC. Delirium-related features of human EEG are generalized theta or delta slow-wave activity with reduced ratio of fast-to-slow band power, reduced mean frequency, and reduced occipital peak frequency (Azabou et al., 2015, van der Kooi et al., 2015). It’s noteworthy to specify here that theta frequency band is investigated differently for humans and rodents. Theta range for humans is 4–7 Hz, while for rodents extends to 5–12 Hz, which overlaps with humans fast alpha rhythm. Similarly, delirium patients expressed a decrease in alpha- and an increase in delta relative power in delirium (Koponen et al., 1989). Low-frequency theta waves are more common in the mild forms of encephalopathy in humans (Young et al., 1992). Our experimental protocol with moderate dose LPS mimics moderate infection-induced encephalopathy. Theta profile in our experiments differed between the hippocampal region and medial prefrontal cortex. While theta frequency was substantially suppressed in hippocampus, there was only temporal mild decrease in mPFC.

### 3.2 Abnormal synchronization

We found also that the LPS-evoked alteration of delta and theta frequency parameters is followed by abnormal synchronization in low frequency ranges. Repetitive, daily, administration of high doses of LPS increases the number of cortical spike-wave discharges in an epileptic strain of rats (Kovacs et al., 2006). The LPS-induced increase in epileptiform activity was not directly correlated with the elevation of the core body temperature, as it is in febrile seizures (Gyorffy et al., 2014, Kovacs et al., 2006). LPS injection triggers synthesis of inflammatory cytokines in the central nervous system that include interleukin (IL)-1β, IL-6, and tumor necrosis factor (TNF)-α (Gatti and Bartfai, 1993, Laye et al., 1994, Pitossi et al., 1997). The acute release of inflammatory cytokines mediates the generation of behavioural responses to LPS known as sickness behaviour (Dantzer, 2001). IL-1β is linked to hyperexcitability underlying febrile seizures (Dube et al., 2005, Heida and Pittman, 2005). IL-1β is strongly pro-convulsive while the endogenous antagonist of IL-1β, IL-1Ra, has robust anticonvulsive effects in bicuculline and kainate-induced epileptic activity (Vezzani et al., 2000, Vezzani et al., 2002), suggesting that IL-1β/IL-1RA balance is important in control of seizure genesis. Despite the extensive studies on the interaction of inflammatory cytokines and epilepsy, the prodromal signs of neuroinflammatory processes in the pathophysiology of epileptic seizures remained underexplored (Curia et al., 2014). A key methodological goal is to identify the early oscillatory features of epileptiform brain activity triggered by systemic inflammation in behaving rats. We showed here that the epileptiform oscillations evoked with LPS application are paralleled by specific theta/delta oscillatory features during the pre-ictal and inter-ictal local field oscillations. Importantly, we observed LPS-generated spike-wave discharges in the hippocampus but not in the cortex. Although EEG can successfully identify and quantify epileptic seizures, there is scarce information about prodromal brain oscillations that precede temporal lobe epilepsy. Here, we explored the subthreshold hyperexcitability features of the local field potentials in the pre-ictal and inter-ictal intracerebral recordings in freely moving rats. We focused our investigation on delta and theta rhythms, which share the same frequency ranges with the epileptiform discharges (Gyorffy et al., 2014). We hypothesize that before brain oscillations enter into abnormal synchronization mode, their frequencies are already altered and resonating in the subsequent epileptiform rates. Therefore, based on the observed prodromal oscillatory profile we can successfully predict the occurrence, severity, amplitude and frequency of the LPS-evoked spike-wave discharges.

## 4 Conclusion

Understanding the interplay between region-specific slow and fast oscillations and brain inflammation could lead to new interventions to minimise the significant impact of acute medical illness on the aging and degenerating brain. The findings described here will allow us to increase the diagnostic accuracy of absence states using reliable electrophysiological features from hippocampal and prefrontal brain regions. We highlight the role of local field oscillations in detecting and evaluating delirium episodes. These data will allow us to elucidate the electrophysiological substrate of accurate delirium diagnosis as well as differential diagnosis with other neurological conditions.

## 5 Experimental procedure

### 5.1 Ethics statement

We conducted our experiments in accordance with directive 2010/63/EU of the European Parliament and of the council of 22 September 2010 on the protection of animals used for scientific purposes and the S.I. No. 543 of 2012, and followed Bioresources Ethics Committee, Trinity College Dublin, Ireland (individual authorization number AE19136/I037; Procedure Numbers 230113-1001, 230113-1002, 230113-1003, 230,113-1004 and 230113-1005), and international guidelines of good practice, under the supervision of Marian Tsanov, who is licensed by the Irish Medical Board (project authorization number: AE19136/P003).

### 5.2 Surgical implantation of recording electrodes

Male, 3–6 months old, Lister-Hooded rats (Envigo, UK) were individually housed for at least 7 days before all experiments, under a 12-h light-dark cycle, provided with water *ad libitum*. Experiments were performed during the light phase. The recording sessions are conducted in a room deprived from external noise and with reduced light (luminosity of 10–15 lux). For our electrophysiological recordings we implanted platinum-iridium electrodes in hippocampus (−3.8 AP, 2.3 ML and 2.4 mm dorsoventral to dura) and medial prefrontal cortex (3.0 AP, 0.5 ML, 3.8 DV). The local field potential (LFP) is sampled at 250 Hz and analysed for the frequency ranges: delta (1–4 Hz), theta (5–12 Hz) and gamma (30–100 Hz). LFP signal frequency analysis is calculated using the short-time Fourier transform of the signal and interpolated into color-coded power spectrograms. After at least one week recovery, subjects were connected, via a 32 channel headstage (Axona Ltd.), to a recording system, which also allowed for animal position tracking. The electrodes was fixed with dental acrylic (Associated Dental, Swindon UK) applied to the anchor screws inserted in the skull. The anchor screw located on the left frontal bone was used as a grounding point.

### 5.3 LPS administration and recording sessions

Bacterial endotoxin (lipopolysaccharide, LPS, *Escherichia coli* O111:B4, Sigma L2630) or sterile saline were administered intraperitoneally (1 mg/kg) by a single injection to 9 Hooded Lister rats. The recordings took place in a square arena (64 × 64 × 25 cm high) situated in the centre of a room with multiple background cues available (surrounding curtains were open). Rats were placed in the open field and 20 mg food pellets (TestDiet, Formula 5TUL) were thrown in every 20 s to random locations within the open field; in this way, animals locomoted continuously, allowing for complete sampling of the environment. Each experimental session was 12 min. A recording session was considered as inactive sessions if the animal locomoted <14 m per 12 min (with average velocity of <2 cm/s) and active if the animal locomoted more than 72 m per 12 min (average velocity of >10 cm/s).

### 5.4 Measurement of local field activity

The field potential (FP) recordings were performed as previously described (Tsanov et al., 2011). The local field potential (LFP) was sampled at 250 Hz and stored for further off-line analysis. LFP signal frequency analysis was done using MATLAB’s Signal Processing Toolbox (MATLAB, Natick, MA) where the power was calculated using the short-time Fourier transform of the signal (hanning window of 2 s, with overlap of 1 s), and interpolated into color-coded power spectrograms. Information was displayed as the magnitude of the time-dependent Fourier transform versus time in a color gradient graph with the maximum corresponding to 0 Db.

### 5.5 Probability of theta frequency and amplitude at different speeds

The LFP instant frequency and amplitude were calculated from Hilbert transform for each frequency band. The data from each band were measured as function of the video sampling frequency. A bivariate distribution of the frequency versus the speed was calculated, where the samples of particular speed value were plotted against the values of particular frequency in the probability color-coded map.

### 5.6 Post-mortem verification of electrode site

At the end of the study, brains were removed for histological verification of electrode localization. The animals underwent transcardial perfusion with 0.1 M PBS followed by 10% formol-saline. The brains were postfixed in 10% formol-saline and then transferred to 25% sucrose overnight. Brain sections (16 µm) were stained according to the Nissl method using 1% toluidine blue, and then examined using a light microscope. Data from brains in which incorrect electrode localization was found (e.g., anterodorsal thalamic nucleus or ventral anterior thalamic nucleus) were excluded.

### 5.7 Statistical analyses

All data were analysed using Prism software (GraphPad Software, Inc, La Jolla, CA). Statistical significance was estimated by repeated measures with one-way and two-way analysis of variance (ANOVA) paired with post hoc Bonferroni test. The probability level interpreted as significant was *p* < .05. All data points are plotted ± sem.
